# Aging and Progression of Beta-Amyloid Pathology in Alzheimer’s Disease Correlates with Microglial Heme-Oxygenase-1 Overexpression

**DOI:** 10.3390/antiox9070644

**Published:** 2020-07-21

**Authors:** Cristina Fernández-Mendívil, Miguel A. Arreola, Lindsay A. Hohsfield, Kim N. Green, Manuela G. Lopez

**Affiliations:** 1 Institute Teofilo Hernando for Drug Discovery, Department of Pharmacology, School of Medicine, Universidad Autónoma de Madrid, 28029 Madrid, Spain; cristina.fernandezm@uam.es; 2 Instituto de Investigación Biosanitaria Hospital de la Princesa, 28006 Madrid, Spain; 3 Department of Neurobiology and Behavior, Institute for Memory Impairments and Neurological Disorders (UCI MIND), University of California, Irvine, CA 92697, USA; maarreol@uci.edu (M.A.A.); lindsay.hohsfield@uci.edu (L.A.H.)

**Keywords:** Heme oxygenase-1, microglia, Alzheimer’s disease, beta-amyloid plaques

## Abstract

Neuroinflammation and oxidative stress are being recognized as characteristic hallmarks in many neurodegenerative diseases, especially those that portray proteinopathy, such as Alzheimer’s disease (AD). Heme-oxygenase 1 (HO-1) is an inducible enzyme with antioxidant and anti-inflammatory properties, while microglia are the immune cells in the central nervous system. To elucidate the brain expression profile of microglial HO-1 in aging and AD-progression, we have used the 5xFAD (five familial AD mutations) mouse model of AD and their littermates at different ages (four, eight, 12, and 18 months). Total brain expression of HO-1 was increased with aging and such increase was even higher in 5xFAD animals. In co-localization studies, HO-1 expression was mainly found in microglia vs. other brain cells. The percentage of microglial cells expressing HO-1 and the amount of HO-1 expressed within microglia increased progressively with aging. Furthermore, this upregulation was increased by 2–3-fold in the elder 5xFAD mice. In addition, microglia overexpressing HO-1 was predominately found surrounding beta-amyloid plaques. These results were corroborated using postmortem brain samples from AD patients, where microglial HO-1 was found up-regulated in comparison to brain samples from aged matched non-demented patients. This study demonstrates that microglial HO-1 expression increases with aging and especially with AD progression, highlighting HO-1 as a potential biomarker or therapeutic target for AD.

## 1. Introduction

Alzheimer’s disease (AD), the most common form of dementia, and other neurodegenerative diseases (NDDs) share common pathological mechanisms such as oxidative stress (OS), low-grade inflammation, mitochondrial dysfunction, metal imbalance and protein aggregation. Many of these alterations also occur during physiological aging, which is considered the major risk factor to develop AD.

Histopathological hallmarks of AD are Tau hyperphosphorylation, which causes the formation of intracellular neurofibrillary tangles (NFT) and extracellular senile plaques as a consequence of misfolded aberrant beta-amyloid (Aβ) peptide deposition [1,2]. Aβ peptide is the result of subsequent cleavages of the transmembrane amyloid precursor protein (APP) [1,3]. Under physiological conditions, APP undergoes proteolysis by α-secretase, leading to a soluble APP fragment involved in the homeostasis of synaptic transmission. However, in AD, APP undergoes abnormal proteolysis, first by β-secretase and then γ-secretase, leading to the formation of Aβ peptides Aβ40 and Aβ42. These monomeric fragments are able to oligomerize, resulting in the formation of the extracellular senile plaques. There are two major categories of AD; familial AD (~2%) and sporadic AD (98%). Familial AD, which appears early in life (40–60 years), is characterized by specific mutations in genes related to APP and presenilin 1 and 2 (PSEN1 and PSEN2 subunits of γ-secretase) that cause an increase in the production of Aβ peptides [3,4,5]. For preclinical AD studies related to Aβ pathology, the 5xFAD mouse is widely used as it expresses human APP and PSEN1 transgenes that engender an AD-like phenotype with rapid and progressive Aβ deposition [6,7].

Microglia are the resident macrophages of the CNS and are responsible for the central innate immune response. These cells have long been considered to have a secondary role in neurodegeneration but are now emerging as central to AD risk [8]. A gradual deterioration, known as immunosenescence, occurs in the immune system with aging, raising susceptibility to diseases like AD. Aged microglia produce more reactive oxidative species and inflammatory cytokines [9]. The overproduction of pro-inflammatory mediators leads to the sensitization of microglia, or age-related microglial priming whereby aged microglia produce an exaggerated, but inefficient, response to inflammatory stimuli. In AD, progressive Aβ plaque formation further contributes to the chronic activation of microglia, which leads to a toxic pro-inflammatory and pro-oxidant environment as a consequence of an unregulated release of cytokines (TNF-α, IL-1β) and nitric oxide (NO), mediators known to damage and kill neurons. Furthermore, in this “activated” state, microglial cells are more phagocytic resulting in greater Aβ clearance. In fact, in AD brain samples, activated microglial cells tend to locate surrounding Aβ plaques in order to clear them. However, this increase in Aβ clearance, rather than being beneficial, seems to favor a non-resolving inflammatory feedforward loop, promoting disease progression [10,11,12].

Heme-oxygenase 1 (HO-1) is the inducible isoform of the heme-degrading enzyme heme-oxygenase. HO-1 converts the pro-oxidant heme group to biliverdin, which rapidly converts into bilirubin and carbon monoxide (CO), all products with high anti-oxidant and anti-inflammatory properties [13,14]. HO-1 can be regulated by many stimuli able to activate numerous cascades and transcriptional factors such as AP1 and NF-kB, which regulate stress and inflammation dependent genes [15,16]. Furthermore, NRF2, the master regulator of antioxidant stress response, has proven to play a major role in HO-1 regulation [17]. Therefore, upregulation of HO-1 is considered a mechanism of cell adaptation to stress with cytoprotective properties, which are related to the gasotransmitter CO. In addition, CO has anti-apoptotic properties and has been proposed as a promising therapeutic strategy for the treatment of inflammatory-related diseases, such as ischemia, experimental autoimmune encephalomyelitis (EAE), or sepsis [18,19,20,21]. However, it has been hypothesized that HO-1 expression increases with ageing in the human normal brain evidenced by a progressive increase in the immunoreactivity for HO-1, both in neurons and neuroglia and in cerebral cortex and hippocampus [22]. Other studies show that in non-demented subjects, HO-1 immunoreactivity was low, though detectable, in various cell types, such as astrocytes, cerebrovascular endothelial cells, or choroid plexus epithelial cells, in different brain regions [23,24]. These results indicate that, during physiological aging, HO-1 could be up-regulated in specific cells and areas of the brain that may be particularly vulnerable to oxidative stress as an adaptive defense mechanism.

Under this framework, we designed this study to analyze the expression pattern of HO-1 with aging and in AD. To address this, we have used the 5xFAD mouse model of familial AD and their age-matched controls, together with postmortem brain samples from non-demented subjects and AD patients. Our results indicate that HO-1 is predominately and progressively overexpressed in microglia with aging, this expression being most remarkable in AD.

## 2. Materials and Methods

### 2.1. Animals

All animal experiments were performed according to animal protocols approved by the Institutional Animal Care and Use Committee (IACUC) at the University of California, Irvine, an American Association for Accreditation of Laboratory Animal Care (AAALAC)-accredited institution (Ethical approval number: AUP-18-139 ). We utilized 5xFAD mice in this study, a model of AD harboring five relevant mutations across two human transgenes, amyloid precursor protein (APP) and presenilin-1 (PSEN1), described in detail elsewhere [6,7] and obtained from the Mutant Mouse Resource and Research Centers (MMRRC; 034848-JAX). WT mice (00664) were obtained from the Jackson Laboratory to maintain both lines on a C57BL/6J background. Mice were housed in groups of up to five animals/cage under 12-hr light/dark cycles, with ad libitum access to vivarium chow and water. For time course experiments, naïve male and female 5xFAD and WT mice were euthanized for investigation at 4, 8, 12, and 18 months (mo). The primers used for the genotyping of the transgenic mice were as follow (5’-3’): PS1 (Fwd.: AATAGAGAACGGCAGGAGCA, Rvs: GCCATGAGGGCACTAATCAT): APP (Fwd.: GCTTGCACCAGTTCTGGATGG, Rvs.: GAGGTATTCAGTCATGTGCT). Animals were euthanized via CO_2_ inhalation and were perfused with cold 1X phosphate buffered saline (PBS). The brains were removed and then fixed in 4% paraformaldehyde (PFA) for immunofluorescent analysis.

### 2.2. Tissue Immunofluorescence

The fixed brains were coronally sliced at 40 µm with a microtome (Leica SM2000R) and, thereafter, stored in glycerol-based solution. For Thio-S staining, sections were washed twice with 1x PBS for 5 min each, followed by dehydration in a gradient of alcohol (100%, 95%, 75%, 50%, 1 × 3 min each). After, the slices were incubated 10 min with Thio-S solution (0.5% Thio-S, 50% ethanol) in the dark, followed by two washes in 50% ethanol (5 min each) and a last wash in PBS (1 × 5 min). Thereafter, the standard for Iba1, GFAP and HO-1 staining was performed. The tissue was washed with 1xPBS (1 × 5 min) and then incubated with the blocking solution (5% donkey-serum in PBS + 0.2% Triton-X100) for 1 h at room temperature. Afterwards, the slices were incubated overnight at 4 °C with the primary antibodies: anti-Iba1 (1:500, ab5076, Abcam), anti-HO-1 (1:500, ab68477, Abcam) and anti-GFAP (1:500, ab4674, Abcam). The tissue was then washed three times in 1x PBS and incubated with the secondary antibodies (1:200, Alexa Fluor 488, 547, or 647, ThermoFisher) for 1 h in the dark at room temperature. Sections were washed with PBS (3 × 10 min), mounted, and images were taken using a SE confocal microscope (SE, Leica) or a Zeiss Zen slide scanner (Axio Scan, Zeiss). Images were analyzed using Fiji software.

### 2.3. RNAscope in Situ Hybridization

RNAscope in situ hybridization was performed in order to establish the cell-specific expression of HMOX1 RNA in microglia, astrocytes or other CNS cell types. Brain slices (40 µm) were mounted and dried overnight at room temperature. Then, the tissue was washed in 1x PBS (1 × 5 min) and baked at 60 °C for 30 min. Afterwards, the slices were dehydrated in a gradient of ethanol (50%, 70%, 100%, 100%, 1 × 5 min) and air-dried for 5 min at room temperature. Once dried, they were incubated with the RNAscope Hydrogen Peroxide for 10 min. Right after, the slices were washed in double distilled water (ddH_2_O, 3 × 5 min). The tissue was then incubated in boiling RNAscope Target Retrieval reagent for 15 min followed by 5 ddH_2_O washes and a last wash with 100% ethanol. Once dried, the slices were incubated with protease III for 30 min at 40 °C and washed in ddH_2_O. The HMOX1 RNAscope probe was added and slices were incubated for 2 h at 40 °C. Once the incubation period was finished, the tissue was washed in Wash Buffer (2 × 2 min). RNAscope amplification reagents were prepared and the slices were incubated consecutively with AMP1, AMP2 and finally AMP3 (30 min at 40 °C followed by two washes in between AMP reagents). HMOX-1 probe was developed with the adequate horse radish peroxidase (HRP) probe (HRP-C2, for 15 min at 40 °C) and afterwards with the correspondent secondary fluorescent antibody (Opal 520, 1:1500) for 30 min at 40 °C. Once the RNAscope protocol was finished, the immunofluorescent protocol was performed as previously described. Finally, sections were covered and the images were taken using a SE confocal microscope (SE, Leica) or a Zeiss Zen slide scanner (Axio Scan, Zeiss).

### 2.4. Human Samples

For analysis of human brains, postmortem cortical tissue from the middle frontal gyrus (BA9 and 46) of non-demented and AD subjects (Table S1) was obtained from the Alzheimer’s Disease Research Center (ADRC), UC Irvine. The protocols for obtaining postmortem brain tissue complied with all federal and institutional guidelines with special respect for donor identity confidentiality and informed consent. Dementia and AD diagnosis were made by a consensus conference using neuropsychological assessment, neurological examination, and medical records following DSM-IV and National Institute of Neurological and Communicative and Stroke-Alzheimer’s Disease and Related Disorders Association criteria, respectively. Neuropathological examination included Braak staging using National Institute on Aging-Reagan criteria [25]. Paraformaldehyde-fixed human samples were cryoprotected via 48 h incubation in 30% sucrose + 0.05% sodium azide and samples were cut into serial sections (30 µm) using a Leica SM2000R freezing microtome. These sections were used for immunostaining with HO-1, Iba1 and GFAP. The protocol used was the same as described above with a slight modification just before mounting the slices. In this case, Sudan-Black solution (0.3% Sudan-Black in 70% ethanol) was incubated for 3 min at room temperature in the dark followed by three washes with ddH_2_O (5 min each). Afterwards, the tissue was mounted, air-dried overnight, and covered.

### 2.5. Image Analysis

In order to determine HO-1 localization in microglia, astrocytes or other cell types, total HO-1 positive particles, HO-1 particles located within Iba1^+^ microglia and HO-1 positive staining co-localized with GFAP^+^ astrocytes was determined. Therefore, the percentage of HO-1 cell localization was estimated. HO-1 positive particles not present within microglia or astrocytes were considered as located in other cell types. To estimate the percentage of microglial cells containing HO-1 (Iba1^+^ microglia positive for HO-1 staining), the total number of microglial cells and the number of microglia positive for HO-1 were determined. For the quantification of HO-1 within microglia, HO-1 mean intensity per Iba1^+^ microglia and per cell was determined. Finally, total microglia co-localized with HO-1 and Iba1^+^ microglia positive for HO-1 located surrounding ThioS^+^ amyloid plaques was estimated in order to determine whether microglial cells overexpressing HO-1 were or were not located nearby amyloid plaques. Fiji software was used for the analysis.

### 2.6. Statistics

Data are presented as mean ± S.E.M. and were analyzed using GraphPad Prism 8.0 software. Two-groups were compared using Student’s *t* test, while multiple comparisons were performed with one-way-analysis of variance (ANOVA) followed by the Tukey post-hoc test. Data distribution was assumed to be normal and significant differences were denoted using the following symbols: * *p* < 0.03, ** *p* < 0.002, and *** *p* < 0.001.

## 3. Results

### 3.1. Expression of Brain HO-1 with Aging in the Alzheimer’s 5xFAD Animal Model

**Figure 1:**
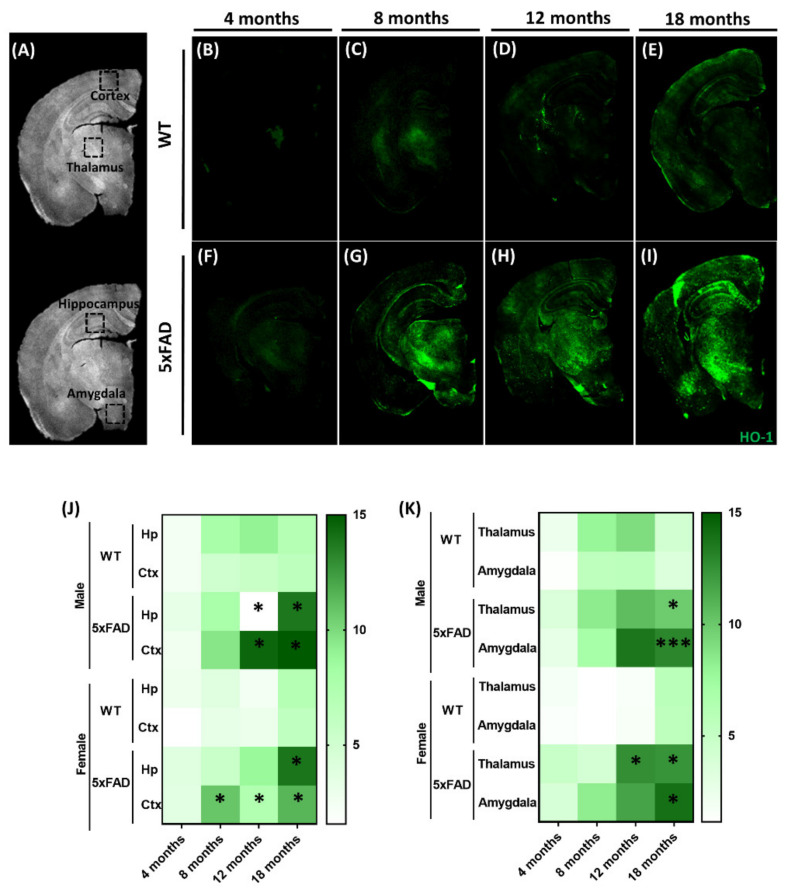
Brain HO-1 expression increases with aging and, preferentially, in 5xFAD mice. (**A**) Whole brain representative images showing the brain regions studied. Representative 10x whole brain images of HO-1 (green) in WT and 5xFAD animals at different time points: 4 months (**B**,**F**), 8 months (**C**,**G**), 12 months (**D**,**H**) and 18 months (**E**,**I**) of age, respectively. Heat maps representing HO-1 mean expression in hippocampus and cortex **(J**) and thalamus and amygdala (**K**) both in male and female WT and 5xFAD mice. Data represent mean ± S.E.M. (*N* = 3–4). Significant differences were considered when: * *p* < 0.03 and *** *p* < 0.001 compared to age-matched WT mice.

We first analyzed the expression pattern of HO-1 in the brain of 5xFAD mice and their littermate WT controls at different ages (4, 8, 12, and 18 months) in the cortex, hippocampus, thalamus, and amygdala (see representative images in Figure 1B–I). Quantification of HO-1 in the selected brain areas (Figure 1A) are depicted as heat maps for the hippocampus and cortex (Figure 1J) and the thalamus and amygdala (Figure 1K). HO-1 expression was significantly increased in the hippocampus and cortex of the 5xFAD mice in comparison to their match WT controls, especially after 12 months of age, both in female and male animals; however, in females, significant increases in HO-1 immunoreactivity were observed in the cortex from eight months of age. In addition, induction of HO-1 was significantly increased in the thalamus and amygdala at 18 months of age in the 5xFAD mice in comparison to WT, both in male and female mice, with the exception of the thalamus of female 5xFAD mice, where significant increase of HO-1 was observed at the earlier age of 12 months (Figure 1K). Taken together, compared to littermate controls, HO-1 was increased in 18 month old 5xFAD mice in all regions of the brain, independently of sex.

### 3.2. Microglia HO-1 Expression Profile in WT and 5xFAD Mice with Aging

**Figure 2:**
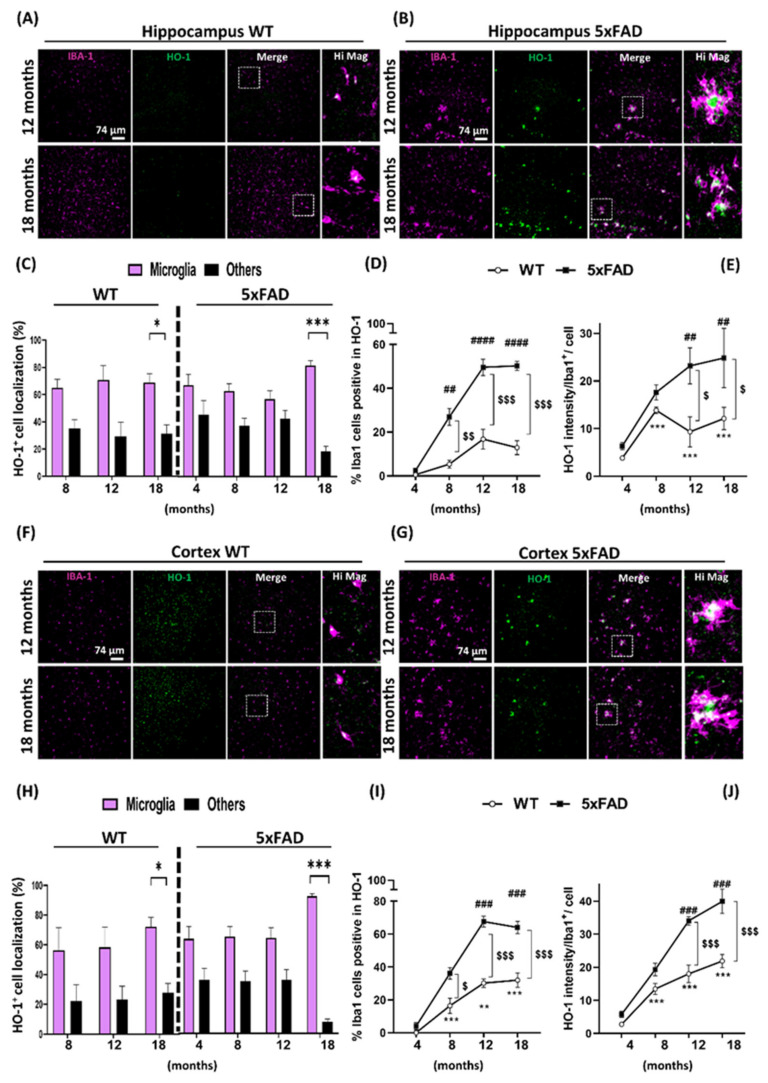
Microglial HO-1 is overexpressed in the hippocampus and the cortex with aging in 5xFAD. (**A**,**B**) 40x representative immunofluorescence images of microglia as Iba1^+^ (magenta) and HO-1 (green) and the merge image (white) in the hippocampus of WT and 5xFAD mice, respectively, at 12 and 18 months of age. (**C**) Specific cell localization of HO-1^+^ staining in microglia (magenta) or other cells (black) expressed as percentage in the hippocampi of WT or 5xFAD mice at the different ages; * *p* < 0.03 and *** *p* < 0.001 compared to the percentage of HO-1^+^ staining in microglia. (**D**) Percentage of total microglial cells expressing HO-1 in WT and 5xFAD mice at different ages. (**E**) HO-1 mean intensity in Iba1^+^ microglial cells was higher in 5xFAD mice and increased with aging. *** *p* < 0.002 compared to 4-month-old WT mice; ^##^ *p* < 0.002, ^###^ *p* < 0.001 compared to 4-month-old 5xFAD; ^$^ *p* < 0.03, ^$$^ *p* < 0.002 and ^$$$^ *p* < 0.001 5xFAD mice compared to aged-match WT controls. 40× representative immunofluorescence images in the brain cortex of WT (**F**) and 5xFAD (**G**) mice at 12 and 18 months old. (**H**) Specific cell localization of HO-1^+^ staining in microglia (magenta) or other cells (black) expressed as percentage in the cortex of WT or 5xFAD mice at the different ages. * *p* < 0.03 and *** *p* < 0.001 compared to the percentage of HO-1^+^ staining in microglia. (**I**) Percentage of total microglial cells expressing HO-1 in WT and 5xFAD cortex at different ages. (**J**) Cortex HO-1 mean intensity in Iba1^+^ microglial cells in WT and 5xFAD mice with aging. ** *p* < 0.002, *** *p* < 0.001 compared to 4-month-old WT mice; ^###^ *p* < 0.001 compared to 4-month-old 5xFAD; ^$^ *p* < 0.03, ^$$$^ *p* < 0.001 5xFAD mice compared to aged-match WT controls. Data represent mean ± S.E.M. (*N* = 4–8).

**Figure 3:**
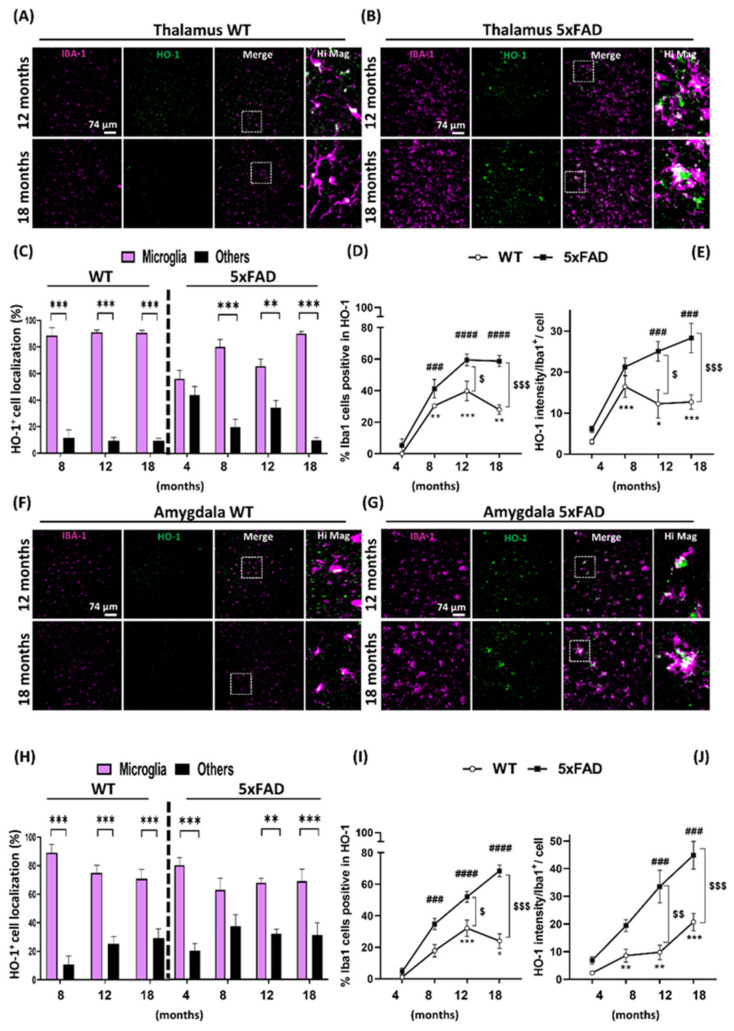
Microglial HO-1 is overexpressed in the thalamus and amygdala with aging in 5xFAD. (**A**,**B**) Representative immunofluorescence images taken with an objective 40x of Iba1^+^ microglia (magenta) and HO-1 (green) and the merge image (white) in the thalamus of WT and 5xFAD mice at 12 and 18 months of age. (**C**) Specific cell localization of HO-1^+^ staining in microglia (magenta) or other cells (black) expressed as percentage in the thalamus of WT or 5xFAD mice at the different ages; ** *p* < 0.002 and *** *p* < 0.001 compared to the percentage of HO-1^+^ staining in microglia. (**D**) Percentage of total microglial cells expressing HO-1 in WT and 5xFAD mice at different ages. (**E**) Amount of HO-1 expressed per Iba1+ microglia cell with aging in WT and 5xFAD mice; ** *p* < 0.002 and *** *p* < 0.001 compared to 4 months WT mice; ^###^ *p* < 0.002 compared to 4-month-old 5xFAD; ^$^ *p* < 0.03 and ^$$$^ *p* < 0.001 5xFAD mice compared to aged-match WT controls. 40x representative immunofluorescence images in the brain thalamus of WT (**F**) and 5xFAD (**G**) mice at 12and 18 months old. (**H**) Specific cell localization of HO-1^+^ staining in microglia (magenta) or other cells (black) expressed as percentage in the amygdala of WT or 5xFAD mice at the different ages; ** *p* < 0.03 and *** *p* < 0.002 compared to the percentage of HO-1^+^ staining in microglia. (**I**) Percentage of microglial cells expressing HO-1 in WT and 5xFAD mice at different ages. (**J**) Mean intensity of HO-1 per Iba1^+^ microglia in WT and 5xFAD mice with aging; * *p* < 0.03, ** *p* < 0.002 and *** *p* < 0.001 compared to 4-month-old WT mice; ### *p* < 0.001 compared to 4-month-old 5xFAD; ^$^ *p* < 0.03, ^$$^ *p* < 0.002 and ^$$$^ *p* < 0.001 5xFAD mice compared to aged-match WT controls. Data represent mean ± S.E.M. (*N* = 6–8).

To further study the pattern of HO-1 expression and localization, we aimed to analyze if HO-1 was being preferentially induced in a particular cell type of the CNS. As HO-1 is an enzyme known to regulate inflammation and knowing that microglia are the cells responsible for the immune innate response, we evaluated HO-1 expression in microglia. For this purpose, co-localization of HO-1 and Iba1, as a microglial marker, was evaluated both in WT and 5xFAD mice, in the hippocampus (Figure 2A,B), cortex (Figure 2F,G), thalamus (Figure 3A,B) and amygdala (Figure 3F,G) at all ages (see Supplementary Figure S1 for earlier ages (four and eight months)). In the hippocampus and cortex, HO-1 was found to be expressed primarily in microglial cells (60–90%) in comparison to other cell types, both in the 5xFAD and WT mice, although statistical significance was only reached at the age of 18 months in both genotypes (Figure 2C,H, respectively).

In the case of the thalamus and amygdala, the cell type in which HO-1 was mostly found expressed, was also the microglia (50–90%). However, compared to hippocampus and cortex, statistical differences between microglia vs. non-microglial cells expressing HO-1, were detected 6 to 10 months earlier in the 5xFAD mice and six months earlier in the WT mice (Figure 3C,H). When we analyzed the percentage of Iba1^+^ cells that were expressing HO-1, we found that this number significantly increased with aging both in WT and 5xFAD mice. However, the percentage of Iba1^+^ microglia expressing HO-1 at 18 months was further increased by around four-fold in the hippocampus (Figure 2D), by around three-fold in the amygdala (Figure 3I), and by around two-fold in the cortex and thalamus (Figure 2I and Figure 3D, respectively) in 5xFAD mice in comparison to WT animals. These results correlated with the amount of HO-1 expressed in microglia cells, measured as the mean intensity of HO-1 in Iba1^+^ cells. The amount of HO-1 in microglia of WT mice increased with age; these differences were significantly evident at ages above 8 months in comparison to four months (2.5–9-fold increase). In the case of 5xFAD mice, microglial HO-1 expression also showed a clear age-dependent increase. In addition, compared to WT mice, microglial HO-1 overexpression in 5xFAD animals was significantly higher at the ages of 12 and 18 months, in all brain areas studied (Figure 2E,J and Figure 3E,J).

We have shown that HO-1 is mostly expressed in microglial cells and this increase is especially evident in 5xFAD mice at the older ages. To see whether at the later stages of AD other glial cells, such as astrocytes, could also be overexpressing HO-1, co-localization studies with GFAP and HO-1 were performed in aged 5xFAD mice. The results of these experiments confirmed that microglia expressed most of the HO-1 found in the different brain areas analyzed, followed by astrocytes and to a lesser extent, in other CNS cells (Figure S2). Furthermore, only in the cortex was the number of astrocytes expressing HO-1 significantly increased in the 5xFAD (17.2%) in comparison to WT (1.2%) mice. Also, the mean intensity of HO-1 in astrocytes was increased in the 5xFAD mice but, statistically significant differences were only observed in the hippocampus (Figure S3). These results further support that HO-1 overexpression is taking place preferentially in microglia and, most importantly, in the AD model.

### 3.3. Localization Analysis of RNA Hmox1 in Glial Cells in Aged 5xFAD Mice

**Figure 4:**
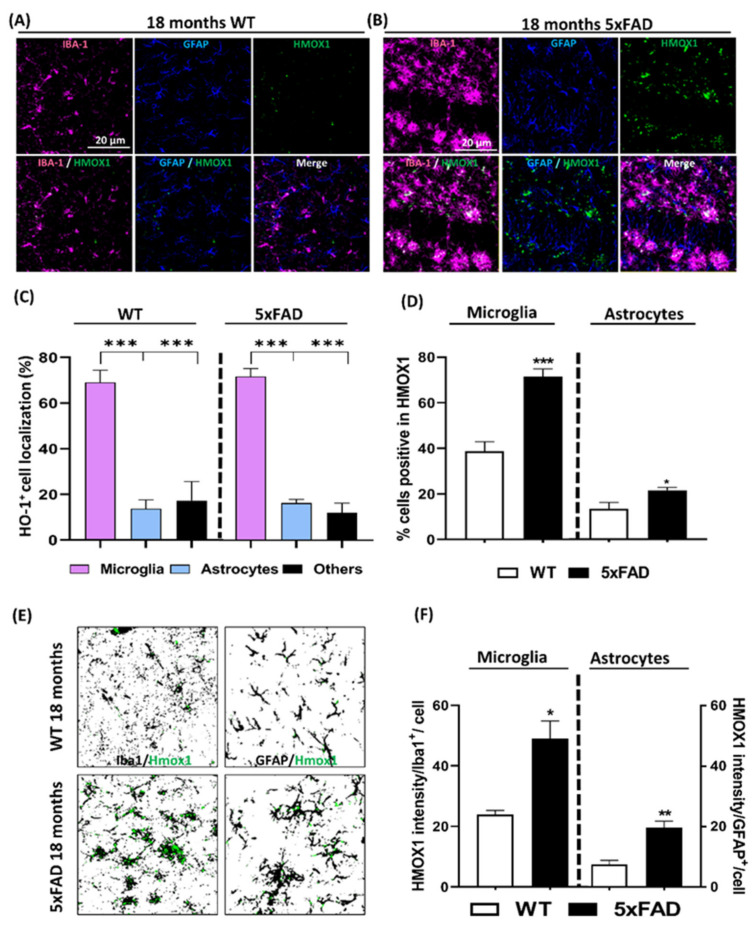
Microglial Hmox1 RNA expression is increased in 18-month-old 5xFAD mice. Representative images of RNAscope immunofluorescence of Iba1^+^ microglia (magenta), GFAP^+^ astrocytes (blue) and HO-1 (green) in 18 months WT (**A**) and 5xFAD animals (**B**). (**C**) Localization of Hmox1 positive signal in percentage in either microglia, astrocytes or other CNS cell types. (**D**) Percentage of total microglia or astrocytes expressing Hmox1 in WT (white column) and 5xFAD mice (black column). There was a significant increase in both microglia and astrocytes expressing Hmox1 in the 5xFAD in comparison to WT mice. However, around 80% of total microglia in 5xFAD animals express Hmox1 while just around 20% of astrocytes express it. (**E**) Representative composite of the percentage of microglia and astrocytes (black) positive in Hmox1 staining (green) (**F**) Hmox1 RNA expression, measured as mean intensity, increases in the 18-month-old 5xFAD microglial cells and astrocytes compared to the WT controls, although Hmox1 mean intensity was higher in microglia than in astrocytes. Significant differences were considered when: *** *p* < 0.001 compared to the percentage of HMOX1^+^ staining in microglia (**C**) and * *p* < 0.03, ** *p* < 0.002 and *** *p* < 0.001 compared to age-matched WT mice (**D**,**F**). Data represent mean ± S.E.M. (*N* = 4–8).

Taking advantage of the RNA in situ hybridization technique, we wanted to corroborate our previous results by detecting Hmox1 RNA in microglia, astrocytes and other cell types in the brain of 18 months old 5xFAD mice (Figure 4B) and their age-matched WT controls (Figure 4A). As represented in Figure 4C, Hmox1 RNA was primarily located in microglial cells (69% in WT, 71.7% in 5xFAD), compared to astrocytes (13.7% in WT and 16.3% in 5xFAD) and other CNS cell types (17.3% in WT and 12% in 5xFAD). In 18-month old mice, the percentage of microglial cells that were positive for Hmox1 was 38.8% in WT mice, while this number was significantly increased to 71.4% in 5xFAD mice (Figure 4D,E). Although there was an increase in the percentage of astrocytes expressing Hmox1 in the 5xFAD, only 27.7% of astrocytes were expressing Hmox1 in comparison to the 71.4% of microglia. Nonetheless, the number of Hmox1 positive astrocytes was significantly higher in the AD model compared to age-matched controls. Similar results were obtained when Hmox1 RNA mean intensity was analyzed in Iba1^+^ cells, the amount of Hmox1 found in microglia was significantly higher in 5xFAD mice compared to age-matched controls. Although the mean intensity expression of Hmox1 was also increased in astrocytes of 5xFAD mice (19 AU), this value was considerably lower than that of in microglial cells (49 AU) (Figure 4F). These results agree with the analysis of HO-1 protein levels and acknowledge that microglia are the cells that are preferentially overexpressing HO-1 in the aged 5xFAD mice.

### 3.4. Localization of Microglia Overexpressing HO-1 Surrounding Aβ Plaques

**Figure 5:**
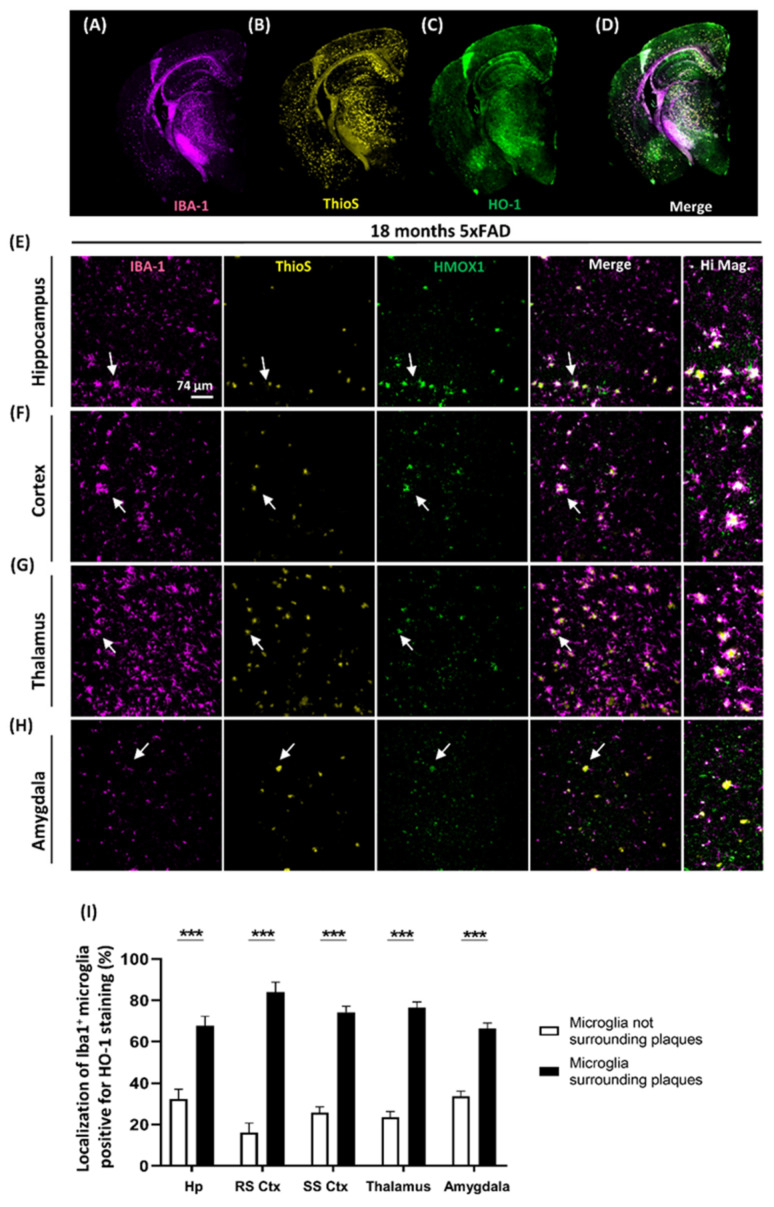
Microglial HO-1 overexpression in 5xFAD mice is preferentially localized surrounding β-amyloid plaques. 10x representative whole brain images (18-month-old 5xFAD mice) of Iba1^+^ microglia (magenta) (**A**), Thio-S (yellow) (**B**), HO-1 (green) (**C**) and the merge channel (white when colocalized) (**D**). 40x representative immunofluorescence images of Iba1^+^ microglia, Thio-S^+^ plaques and HO-1 in 18 months 5xFAD mice in different brain regions: hippocampus (**E**), cortex (**F**), thalamus (**G**) and amygdala (**H**). (**I**) Microglial HO-1 expression occurs mainly (~75–80%) in microglia surrounding plaques in all the different brain regions studied. (*N* = 6). Significant differences were considered when: *** *p* < 0.001 compared to age-matched WT mice. Data represent mean ± S.E.M.

Once we demonstrated that HO-1 was being predominantly expressed in microglial cells, we next decided to determine the sub-localization of these microglia. AD is a proteinopathy characterized by extracellular deposits of β-amyloid plaques. As microglia are the main immune cells in the CNS, we wanted to know if microglia overexpressing HO-1 was located nearby the Aβ plaques. To address this issue, co-localization analysis of microglia stained with Iba1 (Magenta in Figure 5A), β-amyloid plaques stained with Thio-S (yellow in Figure 5B) and HO-1 (Green in Figure 5C) were analyzed in different brain regions of 18 months old 5xFAD mice. As shown in the representative co-localization image of the whole brain (white in Figure 5D) and in the brain regions magnifications (Figure 5E–H), HO-1^+^/Iba1^+^ (white) activated microglia were preferentially located surrounding plaques (yellow) in the 5xFAD mice. As seen in the quantification (Figure 5I), these results indicate that in all the brain regions studied, HO-1 was mainly and significantly induced in microglia surrounding the plaques (~75–80%).

### 3.5. HO-1 Expression Analysis in the Cortex of AD Patients

**Figure 6:**
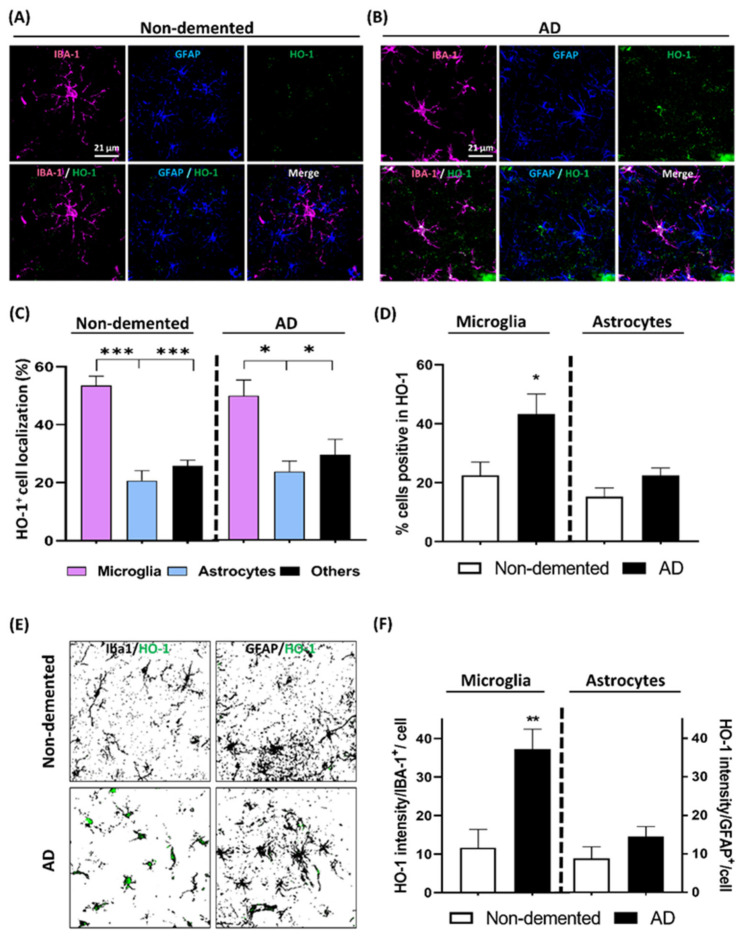
Microglial HO-1 is up-regulated in postmortem human AD cortex samples. Representative confocal images of Iba1^+^ microglia (magenta) and HO-1 (green) in human cortex samples from non-demented (control) subjects (**A**) and AD patients (**B**). (**C**) Quantification of HO-1 specific localization expressed as percentage, in either microglia, astrocytes or other CNS cell types in non-demented and AD patients. Significant differences were considered when: * *p* < 0.03 and *** *p* < 0.001 compared to the percentage of HO-1^+^ staining in microglia. (**D**) Percentage of total microglia and total astrocytes expressing HO-1 in non-demented (white column) and AD patients (black column). There was a significant increase in the number of microglia cells expressing HO-1 in the AD in comparison to non-demented samples. However, there is no significant differences in total astrocytes containing HO-1 between AD and non-demented patients. (**E**) Representative composite of the percentage of total microglia and astrocytes (black) expressing HO-1 (green). (**F**) Microglial HO-1 expression, measured as HO-1 intensity per Iba1^+^ cell, was increased in AD samples compared to non-demented controls. However, astroglial HO-1 expression in AD patients was not significantly increased in comparison to non-demented samples. * *p* < 0.03 and ** *p* < 0.002 compared to non-demented subjects. Data represent mean ± S.E.M. (*N* = 9–10).

To correlate our results in the transgenic AD in vivo model with the human condition, we analyzed postmortem brain cortex samples from age matched AD patients and non-demented controls (Table S1). Iba1, GFAP, and HO-1 immunostaining were performed in order to quantify the expression pattern of HO-1 in microglia and astrocytes, respectively, in the non-demented (Figure 6A) and AD (Figure 6B) brain samples. The percentage of total HO-1 positive staining expressed in microglia cells was significantly higher compared to that expressed in astrocytes or other CNS cells, both in non-demented and AD samples (Figure 6C).

In addition, as seen in Figure 6D, while the percentage of total microglia expressing HO-1 was significantly increased in AD (43.3%) compared to the non-demented patients (22.5%), this was not observed in astrocytes (just around 20% of the astrocytes were expressing HO-1) (Figure 6E). Furthermore, when HO-1 intensity in Iba1^+^ cells was quantified, the expression of this enzyme increased by 3.2-fold in AD samples (Figure 6E). However, in concordance with the total amount of astrocytes expressing HO-1, there was no significant increase in HO-1 intensity in AD astrocytes. Overall, these results indicate that HO-1 is mainly induced in microglia and especially in AD. Notably, these results matched those obtained in the 5xFAD mice, which contributes to the validation of the 5xFAD in vivo AD model.

## 4. Discussion

This study demonstrates that HO-1 is preferentially and progressively up-regulated in microglia with aging and especially in AD. In addition, the majority of microglial cells that overexpressed HO-1 were located surrounding Aβ plaques. These results provide further evidence on the HO-1 expression pattern and distribution in the brain with aging and highlights its importance in AD pathology.

AD is recognized as the most prevalent neurodegenerative disease with still no effective treatment. Therefore, a deeper understanding of the histopathological processes can contribute to finding potential therapeutic targets and biomarkers. We have focused on HO-1 because it is an inducible enzyme related to the control of inflammation and oxidative stress, which are implicated in AD pathology. In the literature, there are contradictory reports related to HO-1 expression in aging and AD [13,14,26]. There are studies, which suggest that NRF2 is downregulated, and thereby also HO-1 [27,28]. On the contrary, other studies show HO-1 upregulation with aging [14], in AD-related animal models [29], and in human AD samples [23,30]. The results of our study are in line with HO-1 upregulation in aging and AD as indicated by increases of this enzyme in WT mice starting at 8 months of age in the thalamus and amygdala and at 12 months of age in the hippocampus and cortex, both in female and male mice. Furthermore, in the 5xFAD model, we also observed a progressive increase in HO-1 expression with aging, this increase being significantly different to WT age-matched controls at the older ages studied (12 and 18 months).

Next, we concentrated on identifying cell specificity of HO-1 overexpression within the brain in aging and AD. We have focused on microglia for several reasons: (i) microglia, the innate immune cells of the CNS, are considered the first line of defense against pathogens or cell damage [10,12,31], (ii) low-grade neuroinflammation is recognized as a hallmark in the pathology of AD and other NDDs [12], (ii) previous reports, indicate that HO-1, under pro-oxidant or pro-inflammatory challenges, increases its expression, mainly in glial cells, and to a lesser extent in neurons [13,14], and (iii) in Dr. Barres Lab RNA-seq database, HO-1 expression was found mainly in microglia and infiltrating macrophages in human and mouse brain [32,33]. Our results show that HO-1 is preferentially located (over 60–90% of total HO-1^+^ staining) within the microglial compartment, in all the brain areas studied, both in WT and 5xFAD animals. Notably, the percentage of microglial cells (Iba1^+^) that were positive for HO-1 progressively increased with age in WT and in 5xFAD animals, although in the AD model such increase was significantly higher from 12 months of age. These results were corroborated with RNA scope, which showed that at 18 months of age, microglial Hmox1 RNA was increased by two-fold in 5xFAD mice compared to their aged matched controls. Furthermore, in human postmortem cortex brain samples from AD patients and non-demented subjects, we also observed that HO-1 was preferentially located in microglia. As observed in the elder 5xFAD animals, in AD patients the percentage of microglial cells expressing HO-1 increased by three-fold compared to non-demented age-matched subjects. Therefore, upregulation of microglia HO-1 could be interpreted as a defense mechanism against the increasing pro-neuroinflammatory and oxidative environment generated with aging and AD progression.

Although our results clearly indicate that HO-1 is being upregulated in microglia, Schipper and co-workers have reported that in the hippocampus and temporal cortex of AD patients HO-1 was found overexpressed in the astrocytes [23], the most abundant cell type in the CNS. Contrary to the results from Schipper and co-workers, in our brain samples HO-1 was significantly upregulated in microglia of AD patients while the number of astrocytes expressing HO-1 in AD samples was not different to non-demented subjects. A possible explanation to this discrepancy could be related to the brain region studied; in fact, in the above-mentioned study of Schipper et al. [23], no increase in GFAP-positive immunolabeled HO-1 astrocytes was detected in the substantia nigra. Interestingly, in the aged 5xFAD model we saw that HO-1 was not significantly increased in astrocytes in all brain regions studied, except for the hippocampus (Figures S1 and S2). In the RNA in situ hybridization analysis, Hmox1 in astrocytes was significantly increased in the 5xFAD brain compared to age-matched WT mice, although it should be noted that the percentage of astrocytes expressing Hmox1 was around three times lower compared to that of microglia. It should be noticed that, although the 5xFAD mouse is commonly used as an AD model, this model might have its limitations, as it is a genetic model with artificial mutations, which are never seen together in individual patients and are related to the familial AD form, being the sporadic AD the most common form [6,7]. Therefore, it would be interesting to further study HO-1 localization and distribution in the hippocampus of AD human samples, of much more relevance than the mouse model and, where according to our results in mice and those from Schipper and co-workers, was the brain region in which HO-1 was significantly increased in GFAP-positive astrocytes.

Activated microglia, characterized by an amoeboid, phagocytic, and pro-inflammatory phenotype, localize surrounding Aβ plaques to induce their clearance. However, this process can result in chronic microglial activation, contributing to disease progression in AD [5,12]. HO-1 has been reported to be upregulated and co-localize with Aβ plaques and neurofibrillary tangles (NFFTs) in AD [23,30,34]. This is in line with our results, which clearly show that around 80% of Iba1-positive cells expressing HO-1 are located surrounding Aβ plaques. The Hmox1 promoter presents response elements to Aβ, which could explain HO-1 upregulation in the microglia surrounding Aβ plaques [13,14]. Microglia HO-1 upregulation could act to reduce the neuroinflammatory response caused by the presence of extracellular Aβ plaques as HO-1 can modulate microglia to facilitate Aβ clearance [34]. However, there is discrepancy, as labile iron, which is related to OS, is one of the end products generated from the activity of HO-1. This could explain the presence of iron deposits localized nearby senile plaques and protein aggregates in AD and other related NDDs [35,36]. In this case, HO-1 overexpression in the brain with aging or in AD could favor the accumulation of iron and ROS, that could contribute to neurodegeneration [37,38,39]. HO-1 upregulation has also been related to the formation of tau oligomers, cognitive decline and synapse aberrations [29,40,41,42] and Aβ plaque formation [34,43]. In contrast, HO-1 inhibition was reported to decrease ROS and improved behavior in AD-animal models [44]. All these findings indicate that HO-1 upregulation could be noxious for the brain. However, there are many other studies that favor HO-1 induction as a therapeutic strategy for AD and other neurodegenerative diseases [45,46,47,48,49]. Actually, there are other CNS-related diseases, such as multiple sclerosis, where HO-1 is found downregulated and this has been hypothesized to be related with relapses of the disease [50]. Therefore, whether HO-1 upregulation is beneficial or detrimental, could depend on the basal expression levels of HO-1. On one hand, in the earlier stages of AD, when HO-1 is still not up-regulated, its induction could have neurotherapeutic effects due to its antioxidant and anti-inflammatory properties derived from its end products biliverdin/bilirubin and CO. Therefore, strategies aiming to induce HO-1, such as hemin, or CO releasers, could be of interest [45,46,47,48,49]. In fact, therapies with multitarget compounds combining Nrf2 activators and CO-releasers have provided promising results in inflammatory-related diseases, such as EAE [18,19,20]. Moreover, in AD models, the beneficial outcomes derived from HO-1 induction against Aβ pathology are linked to CO release [21,46]. As mentioned before, HO-1 up-regulation in microglial cells surrounding Aβ plaques could be related to an anti-inflammatory attempt and a defense mechanism against Aβ-dependent neuroinflammation. Therefore, in this line of work, therapies aimed at further increasing HO-1 expression or the already mentioned CO releasers could be proposed for the treatment of neuroinflammatory-related diseases. However, HO-1 induction has a dark side related to labile iron, another end product of its activity, which leads to OS and cell death when accumulated [14]. Therefore, if HO-1 is already up-regulated, as happens with aging and in the late stages of AD, its induction rather than being beneficial could be deleterious as a consequence of labile iron overload. Thus, therapies aiming to inhibit HO-1 should be considered. In fact, novel HO-1 inhibitors have provided neurotherapeutic effects in vitro and in an in vivo model of AD [39]. Moreover, as one of the main deleterious effects of HO-1 overexpression is the formation of iron deposits, iron chelators should be also considered for AD treatment [51,52,53]. Consequently, a controlled and balanced regulation of HO-1 expression is pivotal in order to achieve beneficial outcomes for the treatment of AD and other age-related diseases.

With these considerations in mind, HO-1 regulation for therapeutic purposes could be problematic; nonetheless, HO-1 upregulation could potentially serve as a biomarker in AD. HO-1 is suggested to be released to the extracellular space by exocytosis or via exosomes [54]. This is in line with studies that reported that HO-1 is up-regulated in cerebrospinal fluid (CSF) or plasma in different inflammatory-related diseases [55,56]. In fact, in PD patients HO-1 was found up-regulated in the saliva, CSF and plasma [57,58,59]. Paradoxically, neither serum nor CSF HO-1 levels were found up-regulated in AD patients, though there was an increase in HO-1 expression levels in the brain. In other studies, performed in sporadic AD patients, HO-1 was found decreased in CSF and plasma [59,60], but this decrease was accompanied by an increase in the HO-1 suppressor (HOS) factor, which could explain the lower levels of HO-1 in the periphery [60]. Therefore, rather than just measuring HO-1, the ratio HO-1/HOS could be considered as a better biomarker for AD.

## 5. Conclusions

HO-1 is progressively upregulated in microglia with aging; this increase is further augmented with disease progression in AD in all areas of the brain studied. Microglia overexpressing HO-1 were preferentially located surrounding the Aβ plaques. Taken together, the results of this study provide new insights on HO-1 expression and distribution in the brain with aging and in AD-progression, that could pave the way to propose HO-1 as a potential biomarker or target for drug development for AD.

## Supplementary Materials

The following are available online at https://www.mdpi.com/2076-3921/9/7/644/s1, Figure S1: Microglial specific HO-1 expression in 4 and 8 months old 5xFAD mice and WT age-matched controls, Figure S2: Microglial specific HO-1 expression in aged 5xFAD mice, Figure S3: Astroglial HO-1 expression in 18-month-old 5xFAD and WT age-matched controls, Table S1: Characteristics of the non-demented subjects and Alzheimer’s disease patients.
